# 

**Published:** 1867-10

**Authors:** 


### BOOKS RECEIVED.

*The Physiology of Man. Alimentation; Digestion; Absorption; Lymph and Chyle.* By AUSTIN FLINT, Jr., M.D., Professor of Physiology and Microscopy in the Bellevue Hospital Medical College, etc. New York: D. Appleton & Co., 443 and 445 Broadway, 1867.

*Is it I: A book for every man.* By Prof. HORATIO ROBINSON STORER, M.D., of Boston, Vice-President of the American Medical Association. Boston: Lee & Shepard, 1867.

*The Physicians' Visiting List for 1868.* Philadelphia: Lindsay & Blakiston.

*Essentials of the Principles and Practice of Medicine: a handy book for students and practitioners.* By HENRY HARTSHORNE, M.D., Professor of Hygiene in the University of Pennsylvania, etc. Philadelphia: Henry C. Lea, 1867.

*Minutes of the Proceedings of the Fourteenth Annual Meeting of the Medical Society of the State of North Carolina, held at Yarboro, N.C., May 15, 1867.*

*A Treatise on Emotional Disorders of the Sympathetic System of Nerves.* By WILLIAM MURRAY, M.D., M.R.C.P., Lond., Physician to the Dispensary and to the Hospital for Sick Children, ect. New York: A. Simpson & Co., 60 Duane St., 1867.

*Clinical Lectures on the Principles and Practice of Medicine.* By JOHN HUGHS BENNETT, M.D., F.R.S.E., Professor of the Institutes of Medicine, and Senior Professor of Clinical

Medicine in the University of Edinburgh, etc. Fifth American from the Fourth London Edition. New York: William Wood & Co., 61 Walker St., 1867.

*Injuries of the Eye, Orbit, and Eyelids: Their immediate and remote effects.* By GEORGE LAWSON, F.R.C.S., Eng., Assistant Surgeon to the Royal London Ophthalmic Hospital, etc. Philadelphia: Henry C. Lea, 1867.

*The Principles and Practice of Disinfection.* By ROBERTS BARTHOLOW, A.M., M.D., Professor of Materia Medica and Therapeutics in the Medical College of Ohio, etc. Cincinnati: K. W. Carroll & Co., 117 West Fourth St.

*Chemistry.* By WILLIAM THOMAS BRANDE, D.C.L., F.R.S., Lond. and Edin., etc., and ALFRED SWAINE TAYLOR, M.D., F.R.S., etc. Second American edition, thoroughly revised. Philadelphia: Henry C. Lea, 1867.

*The Quarterly Journal of Psychological Medicine and Medical Jurisprudence.* Edited by WILLIAM A. HAMMOND, M.D., Professor of Diseases of the Mind and Nervous System in the Bellevue Hospital Medical College, etc.—July, 1867; No. 1, Vol. 1, New York. Terms: $5.00 a year; single copies, $1.50.

*The Tree of Life; or, Human Degeneracy—its nature and remedy, as based on the elevating principle of Orthopathy.* In Two Parts. By ISAAC JENNINGS, M.D. New York: Miller, Wood & Co., 15 Laight St., 1867.

*Elements of Medical Chemistry.* By B. HOWARD RAND, M.D., Professor of Chemistry in Jefferson Medical College. Philadelphia: Y. Elwood Zell & Co., 17 and 19 South Sixth St., 1867.

*The Medical Use of Electricity, with special reference to general electrization as a tonic in neuralgia, rheumatism, dyspepsia, etc.* By GEO. M. BEARD, M.D., and A. D. ROCKWELL, M.D. New York: William Wood & Co., 61 Walker St., 1867.

*The Transactions of the American Medical Association, instituted 1847.* Vol. 18. Philadelphia: Printed for the Association. Collins, Printer, 705 Jayne St. 1867.

popularity. Every intelligent physician is familiar (or should be) with its merits.

***Married.***

In Xenia, Ind., Sept. 22d, 1867, by the Rev. F. W. Keeler, Dr. JUSTIN ROSS, of Williamsport, Ind., to Miss MARIAETTA EGBERT, daughter of Geo. Egbert, M.D., of Xenia, Ind.

***Receipts Acknowledged from:***

D. A. Kittle, $1 25; J. R. Conklin, 1 50; E. Bennett, 3; W. P. Holden, 2; Z. Ball, 2; G. M. Morrow, 2; G. H. Nichols, 2; J. R. Jones, 1 50; H. Steel, 2; J. J. Topliff, 2; C. Palmeter, 2; G. Higgins, 5; W. H. Hess, 5; G. C. Paoli, 2; A. Heavenridge, 2; M. Evens, 2; Cowden & McClelland, 3; Chas. Shepard, 7; J. Walker, 5; H. Gaylord, 5; C. D. Watson, 3; J. P. Anthony, 2; J. V. Hoss, 2; F. E. English, 3; J. W. Hensley, 3; G. W. Allen, 6; D. D. Thompson, 2; Thos. Washburn, 2; J. P. Ross, 2; J. M. Harnett, 3; B. F. Swafford, 5; Lucius Clark, 2; R. F. Henery, 5; J. H. Scott, 2; J. W. Leffingwell, 3; J. J. Brown, 2; F. Hahn, 2; A. F. St. Lure, 2; A. Badenstal, 2; J. M. Sturdivant, 2; A. Morrall, 2 50; W. E. Spelman, 1 50; J. B. Glass, 2; W. P. Sweetland, 2; E. Vogeler, 2; St. Joseph's Med. Society, 2; A. E. Shipherd, 7; A. V. More, 5; J. W. Mitchell, 1 50; J. C. Noyes, 7; T. Bevan, 3; J. M. Evans, 2; C. C. Sprague, 2; C. H. Doneldson, 3; E. W. Boyles, 3; O. B. Adams, 5; P. McAlpen, 3; E. F. Russell, 3; W. S. Hyton, 7; J. F. Daggett, 7; T. B. Dora, 5; A. B. Newton, 2; F. A. Warner, 7; A. Blanchard, 5.

